# Chronic Kidney Disease of Non-traditional Etiology in a Young Man From Central America: Geography, Poverty, and Uncertain Pathophysiology Create a Formidable Medical Challenge

**DOI:** 10.7759/cureus.38876

**Published:** 2023-05-11

**Authors:** Anthony A Milki, Michael Bechara, Susie Q Lew, Adrienne N Poon

**Affiliations:** 1 Department of Medicine, George Washington University School of Medicine and Health Sciences, Washington, USA; 2 Department of Medicine, Division of Hospital Medicine, Virginia Hospital Center, Arlington, USA; 3 Department of Medicine, Division of Kidney Disease and Hypertension, George Washington University School of Medicine and Health Sciences, Washington, USA; 4 Department of Medicine, Division of Hospital Medicine, George Washington University School of Medicine and Health Sciences, Washington, USA

**Keywords:** inequity, dehydration secondary to heat, nephrotoxicity, global health issue, continental central america, environmental exposures, chronic kidney disease of unknown etiology, mesoamerican nephropathy

## Abstract

A man in his early 20s with kidney biopsy-confirmed focal segmental glomerulosclerosis (FSGS) was admitted with one month of nausea and vomiting, intermittent episodes of confusion, shortness of breath, and dysuria. He reported that many people from his native village in Central America, where he harvested sugarcane as a child, have died from kidney disease, including his father and cousin. He believed the source of disease to be agrochemicals found in the village’s water supply. Although FSGS would be a rare manifestation, the patient’s risk factors strongly suggested chronic kidney disease of unknown etiology (CKDu) - also known as Mesoamerican nephropathy (MeN) - a phenomenon he had never previously heard of. He took lisinopril for the last six years to manage his kidney disease. Due to uremic symptoms and abnormal electrolytes, he was initiated on hemodialysis.

## Introduction

Clusters of cases of nephropathies with multifactorial etiologies that are incompletely understood have been reported worldwide. Nephropathies from Central American countries such as El Salvador occurred since the early 1990s [1]. Chronic kidney disease of non-traditional etiology (CKD-nT) occurs most frequently in sugarcane-working men between the ages of 20 and 40 years in the aforementioned regions, geographies that are concurrently characterized by disproportionately high mortality rates from kidney disease [2]. Definitive etiologies for these phenomena have not been determined. However, investigators have noted that in Central American countries such as El Salvador and Nicaragua, more rapid declines in kidney function have occurred during sugar harvest season and hotter weather [3]. These findings have led to research that has identified a variety of possible associated factors that include but are not limited to, chronic heat exposure, dehydration, and agrochemicals [1]. CKD-nT etiology/Mesoamerican nephropathy (CKD-nT/MeN) remains a novel phenomenon that warrants additional investigation. Furthermore, the patient’s kidney biopsy-proven secondary focal segmental glomerulosclerosis (FSGS) (unusual for CKD-nT) pathology, young age of onset, inadequate pharmacotherapy, and poor health literacy make his disease a particularly notable subject of further exploration.

## Case presentation

A Salvadoran gentleman in his early 20s with kidney biopsy-confirmed secondary FSGS was admitted following one month of worsening nausea and vomiting, intermittent episodes of confusion, shortness of breath, and dysuria acutely worsening for 24 hours prior to admission.

**Figure 1:**
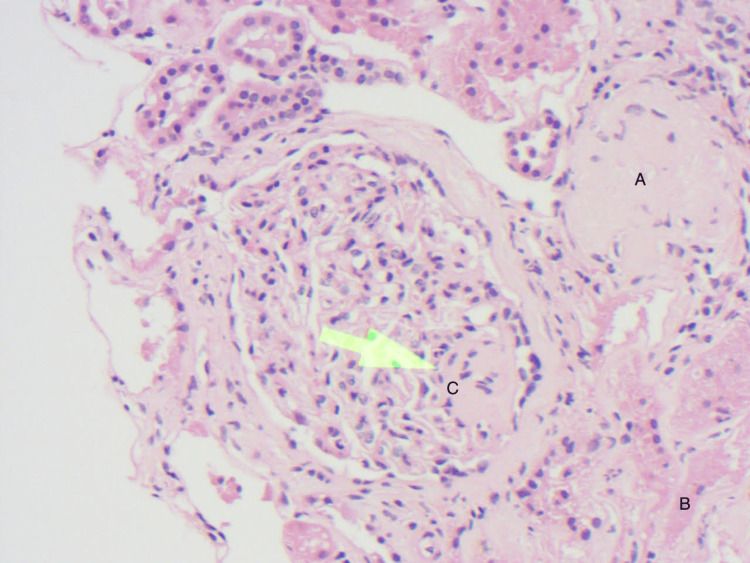
H&E stain: demonstrates globally sclerosed glomerulus (A), mild chronic tubulointerstitial fibrosis involving tubular atrophy (B), and segmental glomerulosclerosis (C).

**Figure 2:**
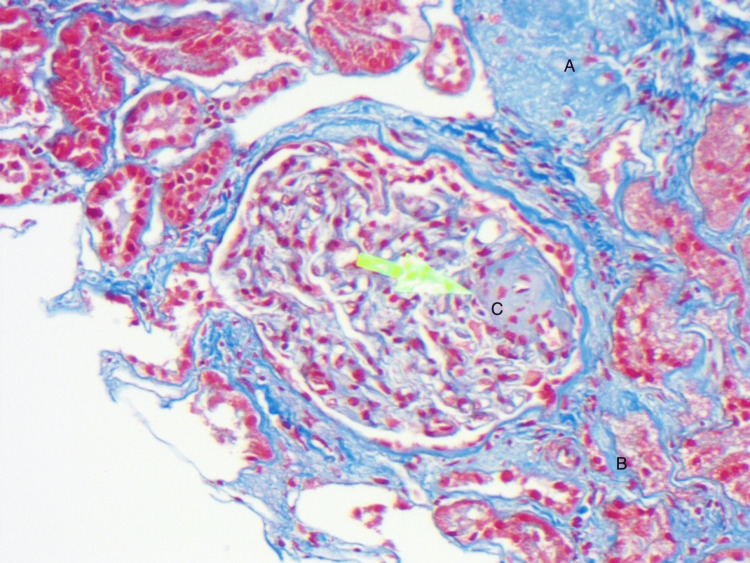
Trichrome stain: demonstrates globally sclerosed glomerulus (A), mild chronic tubulointerstitial fibrosis involving tubular atrophy (B), and segmental glomerulosclerosis (C).

The patient’s declining renal health was observed following a move from Central America to the United States (US). At that time, he was diagnosed with chronic kidney disease by a nephrologist after reporting one year of frequent urinary tract infections, headaches, intermittent flank pain, and decreased appetite in the context of electrolyte abnormalities identified at a routine primary care visit. His creatinine was 1.1 mg/dL with an estimated glomerular filtration rate (eGFR) of 60 mL/min/1.73m^2^, which placed him at stage 2 chronic kidney disease. Since a patient presenting with an elevated serum creatinine of unknown etiology with or without proteinuria requires further evaluation, this patient underwent a kidney biopsy that revealed secondary FSGS (Figures 1, 2). From the age of six until moving to the US at age 14, the patient worked for approximately 10 hours daily in hot temperatures on sugarcane fields to help earn money for his family. He recalls frequently feeling dehydrated due to only intermittent access to water. However, he believed the source of the disease to be agrochemicals found in the village’s water supply. Therefore, he avoided drinking the village’s water, contributing to dehydration. The patient also notably worked as a painter but only began doing so after the presentation of his advanced disease. He denied intravenous drug use, alcohol consumption, HIV infection, and known family history of FSGS.

The patient was prescribed lisinopril at the time of diagnosis. He had decided against the use of corticosteroids for treatment due to potential adverse effects. At that time, the nephrologist also notified the patient that he would eventually require dialysis for kidney failure. Of note, the patient did not receive genetic testing.

The patient reported being adherent with routine outpatient visits every three months to monitor kidney function. He had a serum creatinine measurement of 6.1 mg/dL about half a year prior to his current admission. In the month prior to this admission, the patient began experiencing frequent episodes of nausea and fatigue, with intermittent muscle aches, dysuria, constipation, and shaking across several parts of his body. He also noted a decline in appetite and libido. These symptoms intensified over the week preceding his admission and escalated to the point that he resorted to emergency attention during the 24 hours preceding hospitalization. He also noted three episodes of confusion occurring sporadically over the three months preceding hospitalization. These instances involved a loss of attention during which surrounding individuals were unable to gain his attention for several minutes but subsequently resolved. The intensification of the patient’s symptoms led to the cessation of work as a painter and as a recreational soccer player. When the symptoms exacerbated immediately prior to the emergency department (ED) visit, he began experiencing severe bilateral flank pain, intermittent shortness of breath, left-sided chest pain, and co-occurring tachycardia.

Upon admission, the patient’s vital signs included: blood pressure of 168/116 mmHg, pulse of 109 beats per minute, and SpO_2_ of 99% on room air. His body mass index was 18.12 kg/m^2^. Pertinent labs showed blood urea nitrogen (BUN) of 146 mg/dL, creatinine of 26.7 mg/dL, pCO_2_ of 49.2 mmHg, pH of 7.23, corrected calcium 7.4 mg/dL, phosphorus 10.2 mg/dL, sodium 139 mmol/L, potassium 4.6 mmol/L, chloride 97 mmol/L, and total carbon dioxide 19 mmol/L. His hemoglobin was 8.4 g/dL, platelets 214,000/mm^3^, and white blood cells 9,350 per microliter. Urinalysis demonstrated >500 mg/dL of protein on the dipstick and granular cast on microscopic examination. SARS-CoV-2 polymerase chain reaction (PCR) was negative. Human immunodeficiency Virus (HIV) (SL2) serology was negative. Lisinopril was discontinued in the setting of acute kidney injury.

The patient reported that a large proportion of the inhabitants of his village had kidney disease and that many of them died in their 20s and 30s without receiving hemodialysis. His own father died from kidney disease in his 30s, and several cousins suffered from what the patient described as a similar disease. He was uncertain of any additional details characterizing the kidney disease his peers suffered from but specified that the pathologic trend had anecdotally increased from the previous generation to his own and that his disease onset occurred at a younger age than that of his father (although he does not recall his father’s specific age of onset).

When the diagnosis of FSGS was discussed with the patient during the hospitalization, he was surprised to learn that his disease had a formal diagnosis. He was only aware that his “kidneys had scars on them,” which he stated a previous physician in the US told him in Spanish. According to the patient, the concept of CKD-nT/MeN was never brought up by his healthcare providers. Based on losing young friends and family members to what he believed to be the same disease, he understood that his illness had the potential to become severe and stated that his primary care provider noted he was suffering from gradually deteriorating renal function. However, he did not remember being told that his creatinine level was elevated several months prior to admission and that this signified renal damage. He had been told in the months prior to admission that he would need hemodialysis in the future. Upon severe symptom onset, he told the primary team that he had a suspicion that hemodialysis would likely need to be initiated sooner than he anticipated.

**Figure 3:**
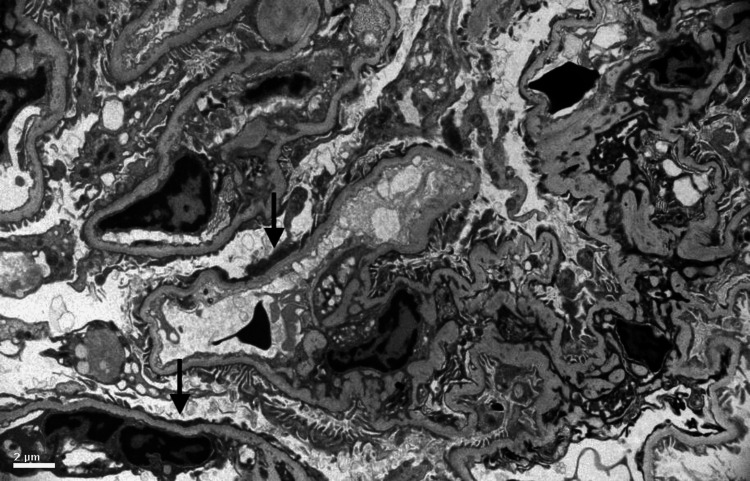
Electron microscopy: taken at 5,000x demonstrates mild patchy foot process fusion (arrows), but otherwise no deposits.

Amid the patient’s lack of confidence in his understanding of his disease history and the absence of accessible electronic health record documentation of the patient’s prior hospitalizations, a literature review conducted by members of the primary team and nephrology service led to the consideration of CKD-nT/MeN as a possible explanation for his constellation of symptoms. As far as the authors are aware, this phenomenon had not previously been explored as a candidate etiology for the patient’s condition. The following insights resulted from discussions between the primary team and the nephrology service. A typical presentation of CKD-nT/MeN includes a young male from an endemic area with a family history of chronic kidney disease, low estimated eGFR, elevated serum creatinine, low level of albuminuria, hypokalemia, hyperuricemia, and urine urate crystals [4]. A kidney biopsy demonstrating tubulointerstitial nephritis confirms the diagnosis. The patient’s clinical findings of severe proteinuria and the absence of hypokalemia and hyperuricemia were not consistent with the classical presentation of this rare diagnosis [5]. His biopsy findings indicated effacement of foot processes on electron microscopy (Figure 3) - a finding characteristic of FSGS [6]. However, the tubulointerstitial involvement also demonstrated in his biopsy was more characteristic of CKD-nT/MeN than it was of FSGS [6]. In addition, a history of intensive agrarian labor with chronic exposure to elevated temperatures in a location with documented associations with CKD-nT/MeN [5] suggested a plausible role being played by the environmental factors posited to underlie the pathophysiology of CKD-nT/MeN, particularly in the absence of common risk factors associated with FSGS [6].

During the hospital stay, he was initiated on hemodialysis using a tunneled dialysis catheter. Many of his symptoms improved and electrolyte disorders corrected or trended toward normal. He was discharged to an outpatient dialysis unit for maintenance hemodialysis three times a week. Follow-up several weeks after discharge confirmed that vascular access was obtained to facilitate long-term hemodialysis. The patient did not require additional emergency medical attention in the following six months after the hospital admission currently being discussed (this was the most recent update obtained by the primary team). He will be evaluated for a kidney transplant in the future.

## Discussion

Awareness of CKD-nT/MeN through education and health literacy may result in increased knowledge of inciting environmental factors, genetic factors, early detection, and treatment [7]. Several cases of adults from Central America presenting to the ED with clinical evidence of renal disease have been published in recent years [8-10]. The narratives are remarkably similar to each other and to the present case: 32-year-old otherwise healthy Salvadoran male with an extensive history of cornfield labor dating back to childhood with a father and uncle who died from renal disease was diagnosed with CKD-nT with subsequent dialysis dependence after an emergency department visit [8]. A 46-year-old Salvadoran male with no risk factors for renal disease with a 14-year history of cotton field and farm work and with a father with advanced renal disease became dialysis-dependent after a CKD-nT diagnosis following an ED visit [9]. Unlike the current case, the above diagnoses occurred later in life and within the frequently observed diagnostic age range of the second to fourth decades [10].

Despite these documented cases, accounting for disease pathophysiology has been a more challenging task for researchers. One explanation for the phenomenon involves frequent heat stress and dehydration due to manual labor in hot climates leading to recurrent renal injury [6]. However, researchers have cast doubt on this prevalent theory, noting that the extent of global warming thus far would be unlikely to account for as drastic an increase in observed CKD/MeN cases in the last several decades, that the impacted individuals are in fact not consistently exposed to hot temperatures, and that the phenomenon has not been seen in other geographies with comparably tropical climates [11]. Separate studies further complicate our understanding of how the disease presents by conflicting with the theory of heat-induced dehydration and indicating a possible role of excessive water intake [12]. Additional data have pointed to pesticide exposure, infection, alcohol intake, and non-steroidal anti-inflammatory drugs (NSAIDs) use as possible contributors to disease development. However, there is little evidence to support these ideas, and a multifactorial pathophysiology that includes roles for water contaminants and dehydration is most likely [13].

There are several possible explanations for the dearth of conclusive information regarding CKD-nT/MeN. In addition to a complex and multifaceted pathophysiology, the disease process has also been consistently observed to impact those living in poverty. It is often noted in rural regions that have historically lacked surveillance programs for renal health and have suffered from limited access to sufficient healthcare, making data collection evasive for researchers [7]. As illustrated above, many impacted individuals are not until they present to the hospital with the advanced renal disease after access to care has improved - in our case, once the patient immigrated from his village in El Salvador to a city in which he received his diagnosis. At that stage, the identification of risk factors is limited to retrospective analysis and preventative care is no longer possible. It is also worth noting that CKD-nT/MeN is a relatively novel and rare diagnosis, with the first cases reported over the last five decades and with formal case series studies delayed until the early 2000s, further explaining the limited understanding the medical field has towards the phenomenon. Educators and healthcare providers in many impacted countries have worked tirelessly to train providers to recognize early signs of kidney disease and to establish screening programs in at-risk populations. There is hope that these efforts will continue to improve CKD-nT/MeN outcomes over time [7].

Characteristic clinical and histopathologic patterns have been observed with relative consistency. However, heterogeneity even in these respects further the challenge of appropriately treating and responding to CKD-nT/MeN. Clinical findings tend to include a constellation of symptoms that have been termed chistata, which is characterized by dysuria, urinary frequency, and chills [1]. Other clinicians and researchers have described muscle weakness and a subjective fever [7]. Our patient, however, did not report or recall experiencing chistata when initially diagnosed with FSGS but was admitted for the current hospitalization with several symptoms overlapping with the above descriptions prior to his initiation of dialysis. Various researchers have posited that histopathologic patterns include glomerulosclerosis, interstitial fibrosis, tubular atrophy, and decreased kidney size in imaging studies [1]. While the specific diagnosis of FSGS is not as frequently attributed to CKD-nT/MeN risk factors, several studies have found sporadic cases of FSGS among those impacted by the phenomenon [14]. In the absence of identified genetic or toxic causes of this patient’s disease and given the previously elaborated biopsy findings with both FSGS and CKD-nT/MeN findings, it is likely that CKD-nT/MeN risk factors contributed to his pathology. The uncertainty surrounding his diagnosis highlights yet another obstacle to appropriately addressing CKD-nT/MeN that patients and providers worldwide experience.

Without an understanding of the risk factors and natural history of CKD-nT/MeN, it becomes impossible to counsel patients in affected regions accurately. Furthermore, even if treatments to slow or reverse the illness progression were identified - which is unfortunately not the case - patients living in regions with limited healthcare access would be unlikely to benefit from any such measures given [15]. The father of the patient we are discussing, for example, reportedly died without having been able to even undergo dialysis treatment for what was known to be advanced kidney disease, and one can only imagine the countless additional lives that have been lost for similar reasons. It is worth noting that CKD-nT/MeN is increasingly being recognized as a health crisis in impacted regions [7]. However, many providers worldwide caring for affected patients have not been educated on its existence, let alone briefed on the ill-defined risk factors that predispose its development. Despite our patient being seen regularly by specialists and primary care providers after immigrating to the United States, it appeared that he had not been told about CKD-nT/MeN. He did not know that severe kidney disease with similar risk factors as he had been observed worldwide, and was under the impression that water toxins alone caused his illness. During his hospital admission, the diagnosis was only discussed after members of the primary team conducted a literature review, and few had previously heard of the illness. The patient had a documented biopsy-supported case of FSGS. Yet he was surprised when the team mentioned that he had an established diagnosis. While he was taking an angiotensin-converting enzyme inhibitor for his FSGS - an appropriate treatment for the disease - he was not aware of alternative first-line or adjunct therapies (cyclophosphamide, calcineurin inhibitors, and mycophenolate mofetil, for example) [16]. Despite the progression of his disease, he did not recall providers revisiting the possibility of initiating corticosteroids, the most effective FSGS treatment [16]. The patient described himself and his family as minimally proficient in the English language around his time of diagnosis and communication barriers are important to account for as a challenge in addressing CKD-nT/MeN for patients and providers.

Our patient’s young age and FSGS diagnosis distinguish his likely case of CKD-nT/MeN from the commonly accepted framework, but he nonetheless exemplifies the unique global and local preventative and diagnostic difficulties - challenges potentiated by healthcare disparities - that underlie the immense amount of suffering that CKD-nT/MeN has inflicted on countless lives. It is therefore crucial for all healthcare professionals to recognize the unique epidemiologic and behavioral risk factors that have been theorized to underlie this disease, and to bear an open mind to the possibility of pathologic and clinical variations to the CKD-nT archetype. This case report is in part intended to increase disease awareness among providers who may not practice in impacted regions. Furthermore, as explanations and curative treatments for those stricken by illness remain elusive, utilizing the knowledge the medical community has amassed may save lives. Reducing work hours for agricultural laborers, allowing time for rest and reducing exposure to sunlight, and providing appropriate hydration from a purified water source in affected regions have been suggested [15].

The patient’s story also teaches us the importance of effective communication. To optimize a patient’s understanding of their illness and involvement in their care, providers treating immigrants who may present with a language barrier in countries like the US can improve compliance with interpretation service guidelines and implement measures such as teach-backs during healthcare encounters, approaches one can imagine may have enhanced our patient’s understanding of his complex medical status [17].

Significant surveillance interventions have been prioritized by various organizations involved in the health of Latin American patients. The Pan American Health Organization (PAHO) has helmed many of these efforts, including a collaboration with the Latin American Society of Nephrology and Hypertension (SLANH) and the US Centers for Disease Control and Prevention (CDC) in 2013 to establish improved surveillance measures in regions impacted by CKD-nT/MeN. PAHO measures have included the use of mortality registries, dialysis center data, survey data, and sentinel surveillance of select individuals working in high-risk occupations - these efforts will intensify with the passage of time and are expected to improve patient outcomes [18].

Although we have focused our discussion on nephropathies clustered in Central America, similar renal pathologies have been observed in other parts of the world; there are cases documented in Sri Lanka, India, Egypt, and Tunisia, where the impacted tend to be poor young men who partake in strenuous agricultural labor [15]. Although this report delineates existing ambiguity towards the once prevailing theory that CKD-nT/MeN is a phenomenon resulting from excessive heat exposure and dehydration, it is important to take this theory seriously in light of the rapidly intensifying warming of global climates. Clearly, much remains a mystery regarding the pathology. However, it is a certainty that the incidence of chronic kidney disease has rapidly increased in the last four decades in regions impacted by CKD-nT [19]. As global temperatures continue to rise precipitously, the incidence of the disease may increase. If that is indeed the case, a failure to advance our fund of knowledge regarding CKD-nT/MeN may mean that our patient’s unusual presentation and remarkably young age, leading to progression to end-stage renal disease may then become the norm [20].

## Conclusions

Physicians globally must be aware of CKD-nT/MeN in the differential diagnosis of kidney disease, particularly when treating patients from impacted regions of the world. It is important to recognize that it is a challenging diagnosis to conclude with certainty for a variety of reasons. While there are previously discussed suspected risk factors for the disease, the pathophysiology remains uncertain. Kidney biopsies are needed to diagnose CKD-nT/MeN, and as our patient’s disease exemplifies, a patient’s history may indicate CKD-nT/MeN while biopsy findings are consistent with renal pathologies not yet understood to be connected to the identified risk factors. Furthermore, most patients are diagnosed with a late-stage chronic kidney disease presentation that requires dialysis, preventing informative tracking of the pathology’s natural history. One can imagine that an incomplete understanding of pathophysiology and inadequate awareness of the disease as a diagnostic option would prevent providers from recognizing risk factors predisposing patients to the development of CKD-nT/MeN, compromising physicians’ pursuit of both prevention and appropriate treatment. This, in turn, would lead to ineffective dissemination of information to patients and their loved ones regarding CKD-nT/MeN and its risk factors.
